# What you learn is more than what you see: what can sequencing effects tell us about inductive category learning?

**DOI:** 10.3389/fpsyg.2015.00505

**Published:** 2015-04-30

**Authors:** Paulo F. Carvalho, Robert L. Goldstone

**Affiliations:** Department of Psychological and Brain Sciences, Indiana UniversityBloomington, IN, USA

**Keywords:** category learning, sequencing effects, interleaved vs. blocked study, comparison, spacing effect

## Abstract

Inductive category learning takes place across time. As such, it is not surprising that the sequence in which information is studied has an impact in what is learned and how efficient learning is. In this paper we review research on different learning sequences and how this impacts learning. We analyze different aspects of interleaved (frequent alternation between categories during study) and blocked study (infrequent alternation between categories during study) that might explain how and when one sequence of study results in improved learning. While these different sequences of study differ in the amount of temporal spacing and temporal juxtaposition between items of different categories, these aspects do not seem to account for the majority of the results available in the literature. However, differences in the type of category being studied and the duration of the retention interval between study and test may play an important role. We conclude that there is no single aspect that is able to account for all the evidence available. Understanding learning as a process of sequential comparisons in time and how different sequences fundamentally alter the statistics of this experience offers a promising framework for understanding sequencing effects in category learning. We use this framework to present novel predictions and hypotheses for future research on sequencing effects in inductive category learning.

Inductive category learning takes place by studying several examples of novel concepts (for instance, several images of a species of snakes). There are several factors that influence the effectiveness of inductive category learning. Amply studied factors include the level of supervision (e.g., Kalish et al., 2011) the delay between seeing an example and receiving information about its category membership (e.g., Maddox et al., 2003; Smith et al., 2014), or the type of categories being learned (e.g., Ashby et al., 1998). In this review we will focus on another important component of inductive category learning—its temporal dynamics. Category learning takes place across time, by studying a series of examples rather than getting all category-relevant information at once, as would be the case with a definition of the category. As such, it is not surprising that the way in which information is organized, i.e., the sequence of events, can have a deep impact on what we learn (e.g., Goldstone, 1996; Schyns and Rodet, 1997), as well as how well we learn it (e.g., Kornell and Bjork, 2008; Wahlheim et al., 2011). There are many theoretically and practically important issues related to sequencing during category learning. Of these, the focus of this review is on the effects of interleaved vs. blocked sequencing. This focus is justified by the current spate of studies focused on this question, and its ease of implementation in classroom and online tutoring contexts.

Analyzing how the sequence of events during category learning shapes learning has obvious relevance for improving learning in different contexts, the classroom being probably one of the most societally impactful ones (Dunlosky et al., 2013). More broadly, we would like to argue that looking at the impact that sequencing factors have on learning has the potential to inform the theoretical views of how learning takes place over time, expanding our understanding of the learning process and its dynamic properties.

## What makes a study sequence better for learning?

The presentation of examples in inductive category learning can be organized in several different ways, such as by level of difficulty (Hull, 1920; Lee et al., 1988; Spiering and Ashby, 2008), variability (Elio and Anderson, 1984; Sandhofer and Doumas, 2008), or similarity relations (Elio and Anderson, 1981; Mathy and Feldman, 2009). Here, we focus on sequences differing not on the properties of the items being shown but on the degree of alternation between categories across successive presentations. For instance, imagine that learners were given the task of learning categories A, B and C, each composed of several items (A_1_, A_2_, B_1_, and so on). Items of each category can be presented in separate blocks (e.g., A1, A2, A3, A4, B1, B2, B3, B4, C1, C2, C3, C4), resulting in a study sequence with infrequent category alternation known as blocked study. On the other extreme of this continuum are sequences with frequent category alternation, known as interleaved study (e.g., A1, B1, C1, A2, B2, C2, A3, B3, C3, A4, B4, C4).

Most research comparing these two study sequences and asking the question of which results in better learning concludes that interleaved study is the most beneficial (e.g., Kornell and Bjork, 2008; Kang and Pashler, 2012; Wahlheim et al., 2012; Zulkiply et al., 2012). For example, Kornell and Bjork (2008) presented learners with several paintings from 12 different artists in either of these two sequences and showed that categorization accuracy for novel paintings in a subsequent transfer task was better following interleaved study (Experiments 1A and 1B). Moreover, following interleaved study participants were also better at determining whether a new painting was painted by a previously studied vs. a new artist (Experiment 2). These results have been shown for different kinds of stimuli and different types of tasks with diverse populations (Taylor and Rohrer, 2010; Wahlheim et al., 2011; Kang and Pashler, 2012; Zulkiply et al., 2012; Birnbaum et al., 2013; Li et al., 2013; Zulkiply and Burt, 2013; Carvalho and Goldstone, 2014b). There is, however, also evidence that blocked study can result in improved or equally effective learning (Kurtz and Hovland, 1956; Goldstone, 1996; Carvalho and Goldstone, 2011, 2014a,b, 2015; Birnbaum et al., 2013; Carpenter and Mueller, 2013; Zulkiply and Burt, 2013; Rawson et al., 2014), which raises the question of what changes with different learning sequences and how do these factors affect learning.

Interleaved and blocked sequences of study differ in several aspects that might contribute to differential learning. For instance, they differ in the amount of time between repetitions of the same category, amount of alternation between categories, and the amount of repetition of parts of successively presented stimuli. The importance of each of these factors and their combinations is not well understood.

One important feature of an interleaved sequence of study is the temporal delay between repetitions of the same category (akin to the “spacing effect” in memory, Ebbinghaus, 1913). Another important factor is the close juxtaposition of stimuli of different categories (akin to the intermixed/blocked effect in discrimination learning, Dwyer et al., 2004). A third factor is the overall “spread” of exemplar presentations throughout the entire learning task (and the associated difference in retention intervals), i.e., in the interleaved condition all categories are equally represented at all points during study, which is not the case in a purely blocked sequence. There is currently no general consensus over which of these properties is the basis for the interleaved study advantage when it is observed. This results in different theoretical proposals based on different cognitive mechanisms with different empirical predictions. Moreover, there are additional contextual factors that have been identified as having a possible modulating effect on the relative benefit of interleaved over blocked study. These factors include the structure of the categories at study (for instance the degree of similarity shared among different categories, Zulkiply and Burt, 2013; Carvalho and Goldstone, 2014b) and the type of task (Rawson et al., 2014; Carvalho and Goldstone, 2015).

An important theoretical and empirical question, thus, is: what makes a specific study sequence beneficial for learning? The objective of this paper is to answer this fundamental question by reviewing the literature examining the effect of interleaved vs. blocked study sequences on category learning. This review will focus on four key factors in the interleaved study advantage for category learning: temporal spacing, temporal juxtaposition, category structure, and retention interval. For theoretical purposes, and to allow a better contrast between the independent benefits of time and space for category learning and the interleaved advantage, in the development of this paper we will use the term interleaved study to refer to alternation of *categories* in opposition to blocked study which is the repetition of categories. Spaced study refers to sequences in which repetitions of specific *items* are spaced over time rather than massed, i.e., verbatim repetitions presented in immediate succession.

### Temporal spacing

The temporal delay between repeated presentations of items has a known effect in retention rates. Overall, if item X is presented several times during study, sequences that include a temporal delay between repetitions of X (spaced study) will result in better memory for that item compared to sequences in which the same number of repetitions of X are presented in immediate succession (massed study). This effect has been repeatedly demonstrated in memory tasks using paired associates and cued recall paradigms (Glenberg, 1976; Glenberg and Lehmann, 1980; Pashler et al., 2007; Cepeda et al., 2009; Delaney et al., 2010).

A related question is whether larger spacing is better than smaller spacing intervals. Larger spacing lags should improve retention because larger spacing increases the difficulty of repeated tests and this increased retrieval difficulty should increase long-term retention (Pyc and Rawson, 2009). Research looking at whether larger temporal lag between repetitions results in improved recall has demonstrated an overall memory benefit with longer spacing intervals (Bloom and Shuell, 1981; Carpenter and DeLosh, 2005; Logan and Balota, 2008; Rickard et al., 2008; Karpicke and Roediger, 2009; Karpicke and Bauernschmidt, 2011). Moreover, recent reviews of the literature indicate that the benefits of increasing the temporal spacing during study depend on the length of the retention interval (Donovan and Radosevich, 1999; Janiszewski et al., 2003; Cepeda et al., 2006). Cepeda et al. (2008) compared a set of temporal lags during study in the context of different retention intervals and noted that when retention interval increases the optimal temporal spacing during study increases as well.

There are several reasons why spacing might benefit long-term retention. One possibility is the increased encoding variability that comes with spacing. When repetitions are spaced in time they are more likely to occur in the context of different items, different emotional/attentional states and different learning states (Glenberg, 1976). This encoding variability may, in turn, result in a higher likelihood of a match between study and test conditions or a memory representation that does not include a particular, narrow context and is, thus, more accessible in the absence of that context (Smith et al., 1978).

Another possibility is that massed presentation increases item familiarity: often times the second encounter with a massed item feels more familiar than a second encounter with a spaced item (Dellarosa and Bourne, 1985). Because of this familiarity sense during massed study, attention to repeated presentation of the same item will be decreased, which will result in less efficient encoding and poorer memory.

Finally, spacing might benefit long-term retention because of increased recall difficulty. Every time a repetition of an item is presented the participant might recall the previous encounter. This recall process is easy in massed study conditions but more effortful with increased temporal lags. Because practice retrieving items from memory improves learning of those items, the more effortful this retrieval (i.e., the longer the delay), the greater is the predicted memory advantage (Bjork and Allen, 1970; Cuddy and Jacoby, 1982; Krug et al., 1990).

The benefits of interleaving different categories have been explained in terms of the benefit of spacing of repetitions (Kornell and Bjork, 2008; Wahlheim et al., 2011). When compared to blocked study, interleaving categories results in more temporal delay between repetitions of the same *category*. Thus, one possible reason why interleaving is often beneficial for category learning is that it increases temporal spacing. Increased temporal delays between repetitions of the same category allows for more forgetting of previous encounters with that category and a more effortful recall of the category properties, which would result in improved encoding of the exemplars and improved test performance, both for memory as well as categorization tasks. Moreover, interleaved study would also result in the same category being studied in the temporal context of a larger number of different categories and across different temporal stages (e.g., beginning, middle and end of the study sessions), resulting in greater encoding variability.

Before we can embrace the possibility that interleaved study benefits learning because of its temporal spacing nature, there is one aspect that needs to be taken into account: it is possible that repeating categories (as is the case in interleaved vs. blocked study sequences) and repeating items (as in spaced vs. massed study sequences) recruit different processes. When the same item is presented a second time there is no variation in its properties. However, when a novel item of a repeated category is presented, it is possible that everything other than the category assignment is different. In this sense, when a category is repeated, the learner's recollection of the previous encounter of the category will often not match the current presentation exactly, unlike what is the case with exact repetitions. This variability may be beneficial because it results in an iterative recall of several exemplars with each novel category encounter (Murray, 1983; Ross, 1984; Ross and Kennedy, 1990; Benjamin and Tullis, 2010; Wahlheim et al., 2014) or in the abstraction of commonalities between repetitions while forgetting the differences (Vlach, 2014), but there is also evidence that similar representations across repetitions result in better memory (Xue et al., 2010). In any case, this iterative recollection process is highly taxing on memory processes, and the resulting memory benefit might be reduced compared to exact item repetitions.

In blocked study sequences this iterative recall process would be less effortful because the item had just been presented when the category is repeated while in interleaved study sequences the greater time lag would result in more effortful recollection of previous items of the same category and better learning (Kornell and Bjork, 2008; Kornell et al., 2010). This conceptualization of the benefit of interleaved study being the result of spacing makes two empirical predictions, based on previous results from studies with item repetitions: (1) the more items are presented in each category, the more effortful recall is and thus learning would be better and (2) the more categories are presented the larger the lag between repetitions of the same category when interleaved, resulting also in more productively effortful recall. This would mean that interleaved study would be more beneficial in situations that include a greater number of categories and items in each category.

Although these hypotheses have not yet been subjected to systematic empirical test, the current evidence from different studies seems to be contrary to the predictions of a theory envisaging larger temporal spacing as the key factor for improved learning with interleaved study. Some studies comparing blocked and interleaved study in the context of category learning have shown interleaved benefits with as few as four categories with only four items presented in each category (e.g., Rohrer and Taylor, 2007, exp. 2; Taylor and Rohrer, 2010) and as many as twelve categories with ten items each (Zulkiply and Burt, 2013). A large number of studies with similar results (better performance after interleaved study than blocked study) include six categories or more with six exemplars or more in each (e.g., Kornell and Bjork, 2008; Kornell et al., 2010; Higgins and Ross, 2011; Wahlheim et al., 2011; Birnbaum et al., 2013). However, there is also evidence for a benefit of blocked study in studies with twelve categories with ten items each (Zulkiply and Burt, 2013), and as few as two categories with two items each (Higgins and Ross, 2011).

Table 1 in Supplementary Materials presents a summary of a survey of 51 studies comparing interleaved and blocked study and how many categories and items in each category were used (for the 36 studies using stimuli organized in categories). For each study a repetition ratio was calculated by dividing the number of items in each category by the total number of categories (if a category contains less items, the category will be repeated less frequently). The median repetition ratio for studies showing a benefit of interleaved study is 1 (range = [0.5, 8]) compared to a median repetition ratio of 1.3 (range = [0.5, 4]) for studies showing a benefit of blocked study. Even though this analysis does not take into account other factors such as the number of repetitions of individual items within each category or the type of test task (memory, problem solving or categorization, for instance) and includes only a small number of studies, it suggests that temporal spacing, as measured by the number of items and categories studied, does not have a large impact over whether interleaving or blocking are more beneficial.

Another possible issue with this formulation of temporal spacing benefits in interleaved study is that, in studies of categorization, learners do not usually recall all of the properties of an individual exemplar, but rather have a biased representation toward some of its features. Research using eye-tracking technology shows that, with the progression of a category learning task, participants progressively attend less to irrelevant properties of the objects and more to the relevant ones (Rehder and Hoffman, 2005; Blair et al., 2009), which would result in a biased representation of the stimuli and, thus, a memory encoding that departs systematically from a faithful representation of the exemplars (Sloutsky and Fisher, 2004; Deng and Sloutsky, 2012, 2013). It is unlikely that when presented with a new exemplar of an old category, participants try to recollect all the features of all of the exemplars seen in that category.

There is however, some evidence that blocked study might result in decreased encoding of immediate repetitions of the same category. Wahlheim et al. (2011) analyzed memory performance for studied items at test based on which position in the study sequence the item had been studied. The results show a decreasing function for blocked study with learners more accurately classifying earlier items into the correct category than later ones. For interleaved study, however, there was no difference in categorization performance across study positions. These results indicate that memory/attentional processes are in play in the relative advantage of interleaved study.

Additionally, Kang and Pashler (2012; see also Mitchell et al., 2008) directly tested the possibility that the benefit of interleaved study is related to greater temporal spacing between repetitions of the same category. In this study, the authors contrasted learners' test performance following a spaced study sequence (in which repetitions of each category were spaced in time but not interleaved—similar to blocked study with added temporal spacing between repetitions) with a blocked study condition and an interleaved condition. The results show that interleaved study results in the best test performance. Moreover, blocked and spaced study result in equivalent test performances. Birnbaum et al. (2013) expanded on these results by comparing test performance in a categorization task following study organized by immediate succession of the same category (blocked contiguous study) with a study condition in which there was an intervening, unrelated, task between each repetition of the same category (blocked spaced study). The results from these experiments showed a benefit of blocked spaced study over blocked contiguous study, while both study sequences were less efficient than an interleaved contiguous version of the sequence (similar to Kang and Pashler, 2012). Interestingly, adding an unrelated task between successive presentations of different categories (interleaved spaced study) resulted in *worse* performance than simple interleaved study and no different than performance following blocked spaced study (for similar results using a discrimination task see Mitchell et al., 2008). The evidence from these studies fits in well with the proposal that there might be some decrease in attentional resources with successive repetitions of the same category which temporal spacing helps break, possibly by introducing a new task that reduces feelings of familiarity. However, temporal spacing, by itself, cannot be the underlying mechanism behind the benefit of interleaved study and is in fact detrimental for category learning with an interleaved study sequence.

In sum, the evidence for temporal spacing as the one factor influencing the advantage of interleaved study is tenuous, although some temporal spacing benefits cannot be ignored for a complete conceptualization of the sequencing effects during category learning. Furthermore, even though blocked study may decrease attentional processing in some situations, it still benefits learning in other situations.

### Temporal juxtaposition

In addition to the possible benefits of temporal spacing, there is another factor that has a substantial impact on learning—temporal juxtaposition. Interleaved and blocked study sequences differ in how closely in time items from the same vs. different categories are experienced. In interleaved study, items from different categories occur temporally closer together than items from the same category while in a blocked study sequence the reverse is true.

Another example of temporal juxtaposition having an influence is the finding that simultaneous presentation of two objects of different categories results in improved discrimination between them when compared to either interleaved or blocked study (MacCaslin, 1954; Williams and Ackerman, 1971; Oakes and Ribar, 2005; Mundy et al., 2007, 2008; Kang and Pashler, 2012; Carvalho and Goldstone, 2014b). For instance, Mundy et al. (2007, Experiment 3) presented learners with pairs of similar morphed faces in a pre-exposure procedure. Each pair could be presented successively in an interleaved fashion or simultaneously. Later, participants completed a same/different task that included the pairs previously presented. Regardless of whether test discrimination was done successively or simultaneously, discriminations were better following simultaneous presentation compared to successive presentation in two ways: (1) two stimuli from the same category are presented at the same time and (2) pairs of the same category are presented on consecutive trials, similar to the blocked sequence of study (for similar results with category learning see Kang and Pashler, 2012; Birnbaum et al., 2013).

In fact, Mundy et al. (2007) proposed that simultaneous presentation is more informative precisely because it reduces the memory constraints in comparing and contrasting to-be-discriminated stimuli. In a follow-up experiment the authors noted that increasing the number of trials in the successive sequence (interleaved study) reduces the numeric difference in discrimination performance following interleaved and simultaneous presentation of items from two different categories, which is consistent with the notion that simultaneous presentation increases the informational value of each trial, hence, more trials in the successive sequence is equivalent to a smaller number of simultaneous trials.

One prominent proposal is that interleaved study differs from blocked study in the way common and differentiating parts of the categories are presented and the same can be said for simultaneous vs. successive presentations (Hall, 1991; McLaren and Mackintosh, 2002; Mundy et al., 2007). In an interleaved sequence, the time lag between repetitions of the *common features between items of the same category* is considerably larger than between repetitions of *discriminating features* (those which distinguish between the categories). This would lead to differences in habituation levels to each part of the stimuli and better discrimination. In a blocked sequence there is no difference in time lag between repetitions of these two parts. Simultaneous presentation results in an intermediate amount of time between successive fixations on the discriminating features. Perhaps these temporal dynamics result in even more habituation to common features, which increases the attentional weighting toward discriminating features and improves discrimination (for similar proposal in the context of category learning see Goldstone, 1996; Kang and Pashler, 2012).

However, before we can fully embrace this possibility, there are two factors that need to be taken into account: (1) there are situations in which successive presentations are more beneficial than simultaneous and (2) in other situations, blocked study results in better performance than interleaved study.

Regarding the possibility that simultaneous presentation is not always better, Lipsitt (1961) showed that when children are asked to discriminate between three similar lights by pressing a different key for each, simultaneous presentation of similar lights (red, pink, and blue) resulted in improved discrimination compared to successive presentation. However, this pattern was reversed for high discriminability stimuli: successive presentation of less similar lights (red, green, and blue) resulted in improved discrimination. Similar results have been shown with children using a delayed match-to-sample procedure (Samuels, 1969), as well as adults discriminating between groups of shapes (Loess and Duncan, 1952), and non-human animals (MacCaslin, 1954). A similar interaction in the opposite direction has also been found with children using a different measure of performance (Williams and Ackerman, 1971).

It is conceivable that in both cases, the advantage results from unequal habituation to discriminating and common features of the stimuli, or a process of contrast that increases the saliency of the discriminating features. In the case of high discriminability stimuli (low similarity categories), less habituation would take place for most of the properties of the stimuli with every new trial (because most of the stimulus would be different in every trial), whereas for low discriminability stimuli (high similarity categories), habituation would occur to a larger degree for the common features. In this sense, perhaps a greater temporal lag between repetitions would help in the case of high discriminability stimuli because it increases memory consolidation which might be important given the greater complexity of encoding all the features of a stimulus—an idea we will come back to in the last sections of this paper.

However, none of these experiments included a blocked successive sequence of study. Can the habituation hypothesis be adapted to account for data showing a learning advantage with the use of a blocked study sequence? Whitman and Garner (1963) had adult participants learn two categories organized by the relational structure of geometrical objects in a figure. The results showed that participants achieved criterion more quickly when stimuli from the same category where presented grouped. Similarly, Carpenter and Mueller (2013) demonstrated that following blocked study of several French words that instantiate the same pronunciation rule, participants were better at identifying that pronunciation rule in novel words in a subsequent multiple-choice test phase. It is hard to imagine how the habituation hypothesis could account for these results. If the same rule was presented over several trials in close succession, participants should have habituated to it and thus it should have been less available for use during test. In much the same way, if during blocked study in Whitman and Garner's (1963) experiments, participants had habituated to the common relation between successive stimuli of the same category, that relation would not have been available and it should take longer to achieve criterion than in an interleaved study sequence.

Another possibility, proposed by Hammer et al. (2008), is that the information available during simultaneous presentation of items from the same category differs quantitatively and qualitatively from the information available if the two stimuli presented simultaneously are from different categories. Hammer et al. (2008), have proposed that simultaneous presentation of items from different categories is most informative when the similarity between the items is high, while the reverse is true for simultaneous comparisons of items of the same category. Specifically, the authors propose that simultaneous study of same category pairs is to some extent always informative because it allows one to infer what properties are not relevant for categorization by determining features that vary across the pairs and further deduce what properties are likely to be relevant. Simultaneous presentation of objects from different categories, on the other hand, may allow inferring which features might be relevant for differentiating objects from different categories, but it is not effective for decisively learning the permitted variability within a category. Moreover, the advantage of simultaneous study of different categories decreases with increasing number of differences between categories, requiring the selection of ideal pairs to allow the isolation of relevant properties across trials (Hammer et al., 2008, 2009a,b).

Evidence for this account comes from experiments with adults and children showing that there is a high level of individual differences in learning proficiency when adults are presented with only few objects from different categories during learning, even if the pairs are selected to be maximally informative (Hammer et al., 2008, 2009b). Moreover, young children (6–10 years) show similar proficiency to adults when learning a complex rule only if presented with pairs of two items from the same category (Hammer et al., 2009a). Hammer and colleagues proposal suggests that while blocked study (similar to simultaneous presentation of same-category items) will foster category learning across a wide range of tasks and developmental ages, interleaved study would require the selection of highly similar exemplars to be effective. In fact, presentation of arbitrary different-category examples that may differ in features relevant for differentiating between categories as well as features that are irrelevant is likely to hinder learning.

In sum, there is some evidence that temporal juxtaposition might play an important role in the benefits of specific study sequences. This evidence led to the proposal of the habituation and discriminative contrast hypotheses. However, these theories are unable to account for all the existing evidence. They cannot account for the learning benefit of blocked study, and accounting for why successive is sometimes better than simultaneous presentation requires resorting to a third variable.

### Category structure

When discussing the importance of temporal juxtaposition there was a variable that stood out as an important factor for whether a study sequence resulted in improved learning or not—category structure. In fact, category structure has been proposed as a key factor modulating sequencing effects in learning. For instance, based on an experiment showing an advantage of blocked study of two categories, Goldstone (1996), proposed that this advantage might be related to the relatively high discriminability between the stimuli of the same category (low within-category similarity). The author proposed that blocked study of the categories would allow participants to notice the subtle but critical within-category similarities that were necessary to learn these high discriminability categories. In agreement with this theory, Carvalho and Goldstone (2014b) demonstrated that by changing only the type of categories presented, participants could show improved learning following interleaved or blocked study. More precisely, interleaved study resulted in better performance for low discriminability categories (categories in which all the stimuli were highly similar, both within and between categories), whereas blocked study resulted in better performance for high discriminability categories (in which all the stimuli were dissimilar, both within and between categories). Similar results have been shown using different types of categories as well (Zulkiply and Burt, 2013).

Although comparing the category structure across different types of stimuli and tasks is unavoidably imprecise, a qualitative survey of the types of categories in the studies surveyed (see Table 1 in Supplementary Materials) indicates that this seems to be the case. All studies showing an interleaved advantage use categories that can be considered to be low discriminability categories, with a large number of shared properties and few discriminating features. Studies showing a blocked study advantage, on the other hand, often involve high discriminability categories in which the objects can be easily discriminated but learning the category rule requires identifying subtle common features.

In sum, category structure seems to have an important modulating effect over which study sequences might result in improved learning and it modulates the effects of different sequences of study (interleaved vs. blocked and successive vs. simultaneous study).

### Retention interval

Another factor that has been demonstrated to play a role in the advantage of interleaved over blocked sequences of study is the time lag between study and test (i.e., the retention interval). It is possible that the benefits of contextual interference promoted by interleaved study are more marked after some retention interval (Shea and Morgan, 1979), although in some cases the benefits also fade way with longer delays (Ste-Marie et al., 2004).

Carvalho and Goldstone (2014a) recently, tested this possibility in the context of different category structures. The results showed an interaction between interleaved and blocked study and the type of category studied (similar to previous results mentioned above). However, this interaction was not modulated by the temporal delay between the end of study and the test.

Conversely, there is some evidence of increased benefit of interleaved study with increased delays in naturalistic settings. For instance, Rohrer et al. (2014b) created an intervention for middle-school math class that included blocked or interleaved study of four types of problems followed by a review session of all the problems studied and a test, either 1 or 30 days after the end of the review session. The results show an overall benefit for interleaved study as well as an increase in the numerical benefit of interleaved study with the increase in retention interval. It is possible, however, that the benefit of retention interval seen here is orthogonal to the relative benefits of different study sequences. It is possible, for example, that greater delays between the end of study and test promote better performance for all sequences of study, the difference being that one characteristic of interleaved study is that it includes a more even distribution of the problems across the entire learning sequence.

Rohrer et al. (2014a) report partial evidence for this account in an experiment comparing interleaved and blocked study of mathematical problems in a naturalistic setting that did not include a review session before test. The results show that the benefit of interleaved over blocked study is smaller for materials studied in earlier blocks, that is, for materials for which the period between last study and test was the longest for blocked study and increases monotonically with decreasing retention intervals between end of blocked study and test. These results indicate that while retention interval may play a role, it is not exclusive so for interleaved sequences.

There is, however, a lack of research exploring the benefits of blocked study using long retention intervals. At first sight this might suggest that whatever blocked advantage is found, it is a short-term one. To optimize learning one needs to take into consideration not only short-term gains but also long-term gains and if blocking is only found as beneficial at short delays, it is perhaps not generally useful in many learning situations. However, regardless of retention interval, the number of studies showing a blocked advantage is low and, as we saw in the previous sections, related to the type of stimuli used.

Why would interleaved study potentiate long-term retention of information to a greater degree than blocked study? One possibility is that learners acquire the information equally well in both the blocked and interleaved sequences, but the introduction of contextual interference, by changing topics or tasks frequently, that accompanies interleaving results in long-term retention of this learning. Nonetheless, it is usually the case that during category acquisition, learners' performance is better for blocked compared to interleaved study (but see Lee, 2012). This may indicate that perhaps participants do learn more in the blocked study sequence but this learning is more transitory, because less cognitive effort is required.

Vlach et al. (2012) presented what can be considered a direct test of this proposal. The authors taught 2 year-old children eight different categories organized around shape, each containing four similar exemplars varying in other properties (color, texture, and size). Different groups of children learned the categories either by studying all the exemplars simultaneously, individually blocked by category, or spaced (similar to the blocked condition but a play time was introduced after each naming trial). No interleaved condition was present in these experiments. Children were tested (1) immediately after learning each category (i.e., after learning the first category, a test session for that category would take place, before teaching the next category), and (2) 15 min later. Simultaneous presentation resulted in the best generalization performance for immediate tests. Interestingly, 15 min later, only children in the Spaced condition were able to generalize the categories learned above chance level. In fact, performance in the spaced condition group did not seem to change across the two time tests, while it decreased considerably for both blocked and simultaneous presentations. This experiment seems to indicate that inserting some contextual interference (in this case by playing between learning trials) resulted in improved long-term benefits. However, there are some procedural details that are worth taking into account in explaining these results. Immediate testing required children to remember only one category (the one they had just studied), while delayed test required children to discriminate between eight categories (unlike for immediate testing, in the delayed test condition all the tests were presented after study of all categories was completed). Accordingly, it is possible that spacing is advantageous for judgments requiring category discriminations, rather than delayed tests *per se*.

There is, however, another possibility that does not involve contextual interference effects. Perhaps performance during study is not related to how much is being learned but with ease of responding (it is easier to always give the same response than changing it on every trial). Furthermore, to the extent that learners are able to identify the relevant components of the task or the categories being learned, long-term retention should be improved, regardless of sequence of study. This would predict that, (1) long-term retention is a function of being successful at a learning task and (2) perhaps if studies using highly discriminable categories, for instance, had employed longer retention intervals, a benefit for blocked study would have been seen as well. Nonetheless, as of now, this remains an open question.

## Implications for category learning

From the previous section one thing is certain: There is not one single factor that seems to explain the advantage in inductive category learning for one sequence over another. In fact, there might not be one single factor influencing study sequencing differences but several, acting in conjunction to shape learning. Additionally, most of the frameworks proposed thus far have envisaged different processes acting when study is interleaved compared to when study is blocked. For instance, the “desirable difficulties” framework (Bjork, 1994) proposes that these desirable difficulties are more present when interleaving and the habituation/discriminative contrast hypotheses (Mundy et al., 2008; Dwyer et al., 2011; Kang and Pashler, 2012; Rohrer, 2012) envision between-category comparison as the principal way to learn categories. However, it is possible to conceptualize a single learning process that would result in study sequencing differences naturally. What this means is that the effect of different sequences on learning efficacy might not be due to the sequence *per se* but the effect that sequencing has on a general-purpose learning process.

Consistent with this view, we have proposed the attentional bias framework (Carvalho and Goldstone, 2014a,b, 2015). One of the fundamental assumptions of this proposal is that during any inductive category learning, learners focus their attention on and encode mostly differences between objects of different categories and similarities among objects of the same category, albeit not necessarily to the same degree. The two main ways for concept learning to proceed are by identifying within-category similarities or between-category differences (Goldstone, 1996). While some accounts of category learning assume that learning must proceed by learning to attend to the features that *discriminate* among the categories being acquired (e.g., the between-category differences), a claim of the attentional bias framework is that categorization can also proceed by developing a positive, stand-alone characterization of a category that highlights the within-category similarities among examples (see also Hammer et al., 2008).

Another assumption is that during category learning, participants weight more heavily information acquired in the previous trial compared to the information acquired longer ago. This assumption is also based on empirical results in the category learning literature. For example, Jones and Sieck (2003) demonstrated the existence of what the authors termed a “recency effect” in category learning—participants are more likely to categorize a novel stimulus into the same category as the previous one if they are similar (see also, Stewart et al., 2002). The basic idea put forward by this research is that recent categorization events play a stronger role in a novel categorization decision than do older events, and categorization decisions are not based on a veridical analysis of the distribution of exemplars across time.

**Figure 1:**
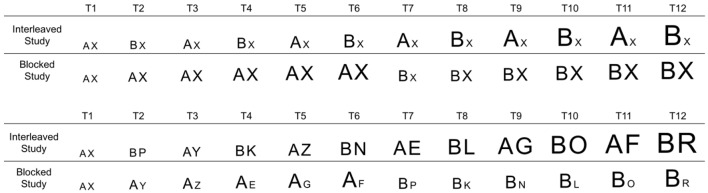
**Schematic representation of the “attentional bias framework” for sequential order effects in category learning**. Objects are represented by pairs of letters, where each letter refers to a feature. There are two categories; one characterized by the presence of Feature A and the other Feature B, while the category exemplars have other features as well. The top panel represents categorization of two low discriminability categories (both categories share the X feature), while the bottom panel represents categorization of two high discriminability categories in that the two categories do not share any feature between them. The size of each letter reflects the attention paid to it.

Taking into consideration these assumptions, one can hypothesize category learning as a continuous stream of category decisions in which attention is successively directed toward relevant similarities/differences by successive comparisons between the current trial and the recollection from the previous one(s). On each learning trial, the learner evaluates similarities and differences between the current stimulus and the recollection they have of the previous one(s), as well as the correct category assignment of the previous exemplar and the current one. If the previous object is similar to the current one and they belong to the same category, attention will be directed toward similarities. However, if they belong to different categories, attention will be directed toward differences. In this way, *across time* attention will be more and more biased toward relevant within-category similarities and between-category differences. This will in turn affect category representation, which will affect category encoding and recollection. With each new trial, the relevant properties will be progressively better encoded while irrelevant ones will be poorly or not encoded at all (see Figure 1 for a schematic representation of this proposal across different category structures).

When categories are studied interleaved, the number of transitions between objects of different categories is highly frequent, which will result in attending to differences between categories in most trials by the process described above (potentially also encoding within-category similarities that are repeated close in time). In the same way, when categories are studied blocked, the likelihood of a within-category transition is high, which will increase attention toward within-category similarities by the same process. This, in itself, cannot explain why interleaved study is more beneficial under some situations than others, of course. To do that, we need to also take into account the appropriateness of each of these two category-learning processes (identifying between-category differences and identifying within-category similarities) for the task at hand.

What this framework posits is that in situations that require learning differences between categories, interleaved study will accelerate learning by promoting encoding of exactly these properties of the objects. On the contrary, for situations that require learning similarities within categories, blocked study will accelerate learning by promoting encoding of these similarities. It is important here to note that what the sequence of study is doing, according to this framework, is changing the relative frequency/statistics of different temporally proximate similarities and differences. This, in turn, affects the normal learning process resulting in differential encoding of stimuli properties.

What is the evidence for this framework? For one, these results are consistent with the research showing that interleaved study improves learning of low discriminability categories (where finding differences is key) while blocked study improves learning of low similarities categories (where finding similarities is key) (Zulkiply and Burt, 2013; Carvalho and Goldstone, 2014b). It is also consistent with most of the research showing benefits of interleaved study for low discriminability categories (e.g., Kornell and Bjork, 2008; Taylor and Rohrer, 2010; Kang and Pashler, 2012), and blocked study benefits for high discriminability categories (e.g., Kurtz and Hovland, 1956; Goldstone, 1996; Carpenter and Mueller, 2013). This might also explain why simultaneous presentation benefits learning of low discriminability categories but not high discriminability categories (e.g., Loess and Duncan, 1952), if one construes simultaneity as a limiting case of extreme temporal proximity.

However, the implications of this framework go beyond the properties of the categories. Any situation that changes the relative importance of differences between categories vs. similarities within categories should show similar results. Evidence for this comes from research showing that interleaved study benefits active learning situations while blocked study benefits passive learning situations (Carvalho and Goldstone, 2015), A similar interaction between the type of study sequence and whether examples of different categories are presented with a definition or not has also been found (Rawson et al., 2014). In this study when learners where given a definition of each concept and studied examples of the different concepts, blocked study resulted in better later classification of new examples. However, when no definition was given along with the examples, interleaved study resulted in better classification of new examples during test (Rawson et al., 2014). Importantly, changes in the relative importance of differences between categories vs. similarities within categories are not limited to different encoding situations but can be created by different testing situations as well. For example, Carvalho and Albuquerque (2012) demonstrated that interleaved study resulted in better performance in a test task that required discrimination between pairs of stimuli but not in a test task that did not require knowing these discriminative properties (see also de Zilva and Mitchell, 2012).

What happens in situations where the statistics of the sequence do not target the properties relevant for the particular learning situation at hand? One possibility is that learners will try to memorize individual exemplars instead of noticing these properties (for a detailed description of this possibility see Carvalho and Goldstone, 2014a). Evidence of this comes from studies showing that changing the sequence to include factors known to improve memory result in learning benefits in situations that do not target the relevant properties in the learning situation at hand (e.g., blocked study of low discriminability categories) but deter learning in situations that target such properties (e.g., interleaved study of low discriminability categories) (Kang and Pashler, 2012; Birnbaum et al., 2013).

## Predictions and hypothesis for future research

The theory proposed here makes three main predictions: (1) whether a study sequence is beneficial for learning or not depends on the locus of categorization difficulty or the test situation, (2) biasing the object comparison underlying category learning toward the difficulties involved in learning the specific set of categories results in improved learning, and (3) long-term representations of the categories are biased toward the properties more attended to during study, whether those benefit learning or not.

Some of these predictions have already been tested empirically. For instance, as mentioned before, interleaved study benefits learning low discriminability categories while blocked study is beneficial when learning high discriminability categories. These findings are in agreement with predictions (1) and (2). However, there is currently no direct evidence for prediction (3). Future research is needed to test whether the long-term benefits of interleaved study compared to blocked study are related to poor encoding in the absence of interference or, as proposed here, with the match between what was learned during study (the encoded representation of the categories) and the properties of the test phase. Perhaps following blocked study learners have a better memory representation of the similarities within categories, which would allow them to use these features more efficiently if they later become relevant (which is not the case in the studies conducted so far).

This possibility is particularly relevant for considering the consequences of blocked vs. interleaved presentations for transfer to new categorizations. If the representations for two simultaneously acquired categories emphasize their discriminating features, as would be expected with interleaved study, then transfer to a situation with partially new categories might be poor. If a math student learns to discriminate between quadrilaterals that are rhombuses vs. those that are not, the feature “four equal length sides” becomes discriminatively critical. This could lead to poor transfer if the student must subsequently learn to distinguish parallelograms from quadrilaterals that are not parallelograms because this feature is no longer discriminative for the new categorization. By comparison, category learning that emphasizes all of the features shared by category members, as expected from blocked study, might be expected to lead to better transfer. The feature “two pairs of parallel lines” is common to all rhombuses, and would likely be extracted from blocked study, even though it does not serve to discriminate rhombuses from other parallelograms. The general prediction is that if one is uncertain about what future categorizations are needed, blocked study may be a safer, less presumptive approach. In any case, understanding what properties of the stimuli are more efficiently encoded with different sequences of study would allow for general predictions about learning beyond how it benefits specific situations tested.

Moreover, the relative benefits of interleaved and blocked study as envisioned here are dependent on a myriad of other variables, which have not been tested yet. For instance, comparing successive objects is likely to be affected by the overall variability found in the category space, and the type of category space used, for instance whether rule-based or information-integration categories are being studied (Ashby et al., 1998). Another relevant factor is the number of verbatim repetitions vs. category repetitions during category learning. Perhaps the benefits of interleaved study can be offset by increasing the number of new exemplars vs. exact exemplar repetitions during study because novel objects require longer inspection and encoding effort than repetitions. Alternatively, it is possible that the more varied and different the stimuli in a category are, the larger the benefit of blocked study would be.

Additionally, identifying between-category differences as proposed here requires one to be able to notice (and ignore) within-category similarities. Perhaps the optimal way to learn a category is to start by blocking each category, identifying relevant similarities, and then transitioning to interleaved study, using the identified similarities to bootstrap the identification of differences (Gentner and Markman, 1994). This is going to be particularly beneficial with novel categories or with young children because the space of categorization is unknown and blocked study allows for the identification of relevant category groups, which will support identifying differences later on.

Finally, the research presented here raises the broader point about category learning as a process across time and the importance of considering sequential factors in theories of category learning as well as formal models of category learning. Most of the current models of categorization assume that learners have access to the entire category space when making novel category decisions (Nosofsky, 1986; Kruschke, 1992; Ashby et al., 1998; Love et al., 2004) and the process through which differential encoding of stimulus features takes place across learning is not fully understood. One of the important steps for a complete understanding of how category learning takes place across time is the development of complete formal models that include temporal dynamics such as the sequencing factors reviewed here, capable of generating new hypothesis and testable predictions (see for example Stewart et al., 2002). It will also be important to extend the efforts in employing this research to applied situations, for example in schools. Taking advantage of formal models and clear quantitative predictions would allow suggestions for improved learning beyond crude one-solution-fits-all recommendations. Understanding the learning situation as a whole allows one to suggest not a single learning strategy but rather provides the flexibility of knowing how to choose between different learning strategies.

### Conflict of interest statement

The authors declare that the research was conducted in the absence of any commercial or financial relationships that could be construed as a potential conflict of interest.

## References

[B1] Ashby F. G. Alfonso-Reese L. A. Turken A. U. Waldron E. M. (1998). A neuropsychological theory of multiple systems in category learning. Psychol. Rev. 105, 442–481. 10.1037/0033-295X.105.3.442 9697427

[B2] Benjamin A. Tullis J. (2010). What makes distributed practice effective? Cogn. Psychol. 61, 228–247. 10.1016/j.cogpsych.2010.05.004 20580350 PMC2930147

[B3] Birnbaum M. S. Kornell N. Bjork E. L. Bjork R. A. (2013). Why interleaving enhances inductive learning: the roles of discrimination and retrieval. Mem. Cogn. 41, 392–402. 10.3758/s13421-012-0272-7 23138567

[B4] Bjork R. A. (1994). Memory and metamemory considerations in the training of human beings, in Metacognition: Knowing about Knowing, eds Metcalfe J. Shimamura A. P. (Cambridge, MA: MIT Press), 185–205.

[B5] Bjork R. A. Allen T. W. (1970). The spacing effect: consolidation or differential encoding? J. Verbal Learn. Verbal Behav. 9, 567–572 10.1016/S0022-5371(70)80103-7

[B6] Blair M. R. Watson M. R. Walshe R. C. Maj F. (2009). Extremely selective attention: eye-tracking studies of the dynamic allocation of attention to stimulus features in categorization. J. Exp. Psychol. 35, 1196–1206. 10.1037/a0016272 19686015

[B7] Bloom K. C. Shuell T. J. (1981). Effects of massed and distributed practice on the learning and retention of second-language vocabulary. J. Educ. Res. 74, 245–248 10.1080/00220671.1981.10885317

[B8] Carpenter S. K. DeLosh E. L. (2005). Application of the testing and spacing effects to name learning. Appl. Cogn. Psychol. 19, 619–636 10.1002/acp.1101

[B9] Carpenter S. K. Mueller F. E. (2013). The effects of interleaving versus blocking on foreign language pronunciation learning. Mem. Cogn. 41, 671–682. 10.3758/s13421-012-0291-4 23322358

[B10] Carvalho P. F. Albuquerque P. B. (2012). Memory encoding of stimulus features in human perceptual learning. J. Cogn. Psychol. 24, 654–664 10.1080/20445911.2012.675322

[B11] Carvalho P. F. Goldstone R. L. (2011). Sequential similarity and comparison effects in category learning, in Proceedings of the 33rd Conference of the Cognitive Science Society, eds Carlson L. Holscher C. Shipley T. (Austin, TX: Cognitive Science Society), 2977–2982.

[B12] Carvalho P. F. Goldstone R. L. (2014a). Effects of interleaved and blocked study on delayed test of category learning generalization. Front. Psychol. 5:936 10.3389/fpsyg.2014.00936 PMC4141442 25202296

[B13] Carvalho P. F. Goldstone R. L. (2014b). Putting category learning in order: category structure and temporal arrangement affect the benefit of interleaved over blocked study. Mem. Cogn. 42, 481–495. 10.3758/s13421-013-0371-0 24092426

[B14] Carvalho P. F. Goldstone R. L. (2015). The benefits of interleaved and blocked study: different tasks benefit from different schedules of study. Psychon. Bull. Rev. 22, 281–288. 10.3758/s13423-014-0676-4 24984923

[B15] Cepeda N. J. Coburn N. Rohrer D. Wixted J. T. Mozer M. C. Pashler H. (2009). Optimizing distributed practice: theoretical analysis and practical implications. Exp. Psychol. 56, 236–246. 10.1027/1618-3169.56.4.236 19439395

[B16] Cepeda N. J. Pashler H. Vul E. Wixted J. T. Rohrer D. (2006). Distributed practice in verbal recall tasks: a review and quantitative synthesis. Psychol. Bull. 132, 354–380. 10.1037/0033-2909.132.3.354 16719566

[B17] Cepeda N. J. Vul E. Rohrer D. Wixted J. T. Pashler H. (2008). Spacing effects in learning: a temporal ridgeline of optimal retention. Psychol. Sci. 19, 1095–1102. 10.1111/j.1467-9280.2008.02209.x 19076480

[B18] Cuddy L. J. Jacoby L. L. (1982). When forgetting helps memory: an analysis of repetition effects. J. Verbal Learn. Verbal Behav. 21, 451–467 10.1016/S0022-5371(82)90727-7

[B19] Delaney P. F. Verkoeijen P. P. J. L. Spirgel A. (2010). Spacing and testing effects: a deeply critical, lengthy, and at times discursive review of the literature. Psychol. Learn. Motiv. 53, 63–147 10.1016/S0079-7421(10)53003-2

[B20] Dellarosa D. Bourne L. E. (1985). Surface form and the spacing effect. Mem. Cogn. 13, 529–537. 10.3758/BF03198324 3831710

[B21] Deng W. Sloutsky V. M. (2012). Carrot-eaters and moving heads: salient features provide greater support for inductive inference than category labels. Psychol. Sci. 23, 178–186. 10.1177/0956797611429133 22228644

[B22] Deng W. Sloutsky V. M. (2013). Effects of training on category learning, in Proceedings of the 35th Annual Conference of the Cognitive Science Society eds Knauff M. Pauen M. Sebanz N. Wachsmuth I. (Austin, TX: Cognitive Science Society), 2166–2171.

[B18a] de Zilva D. Mitchell C. J. (2012). Effects of exposure on discrimination of similar stimuli and on memory for their unique and common features. Q. J. Exp. Psychol. 65, 1123–1138. 10.1080/17470218.2011.644304 22417278

[B23] Donovan J. J. Radosevich D. J. (1999). A meta-analytic review of the distribution of practice effect: now you see it, now you don't. J. Appl. Psychol. 84, 795–805 10.1037/0021-9010.84.5.795

[B24] Dunlosky J. Rawson K. A. Marsh E. J. Nathan M. J. Willingham D. T. (2013). Improving students' learning with effective learning techniques: promising directions from cognitive and educational psychology. Psychol. Sci. Public Interest 14, 4–58 10.1177/1529100612453266 26173288

[B25] Dwyer D. M. Hodder K. I. Honey R. C. (2004). Perceptual learning in humans: roles of preexposure schedule, feedback, and discrimination assay. Q. J. Exp. Psychol. B Comp. Physiol. Psychol. 57, 245–259. 10.1080/02724990344000114 15204109

[B26] Dwyer D. M. Mundy M. E. Honey R. C. (2011). The role of stimulus comparison in human perceptual learning: effects of distractor placement. J. Exp. Psychol. Anim. Behav. Process. 37, 300–307. 10.1037/a0023078 21500928

[B27] Ebbinghaus H. (1913). Memory: a Contribution to Experimental Psychology. New York, NY: Teachers College, Columbia University 10.1037/10011-000

[B28] Elio R. Anderson J. R. (1981). The effects of category generalizations and instance similarity on schema abstraction. J. Exp. Psychol. Hum. Learn. Mem. 7, 397–417 10.1037/0278-7393.7.6.397

[B29] Elio R. Anderson J. R. (1984). The effects of information order and learning mode on schema abstraction. Mem. Cogn. 12, 20–30. 10.3758/BF03196994 6708807

[B30] Gentner D. Markman A. B. (1994). Structural alignment in comparison - no difference without similarity. Psychol. Sci. 5, 152–158 10.1111/j.1467-9280.1994.tb00652.x

[B31] Glenberg A. M. (1976). Monotonic and nonmonotonic lag effects in paired-associate and recognition memory paradigms. J. Verbal Learn. Verbal Behav. 15, 1–16 10.1016/S0022-5371(76)90002-5

[B32] Glenberg A. M. Lehmann T. S. (1980). Spacing repetitions over 1 week. Mem. Cogn. 8, 528–538. 10.3758/BF03213772 7219173

[B33] Goldstone R. L. (1996). Isolated and interrelated concepts. Mem. Cogn. 24, 608–628. 10.3758/BF03201087 8870531

[B34] Hall G. (1991). Perceptual and Associative Learning. New York, NY: Oxford University Press 10.1093/acprof:oso/9780198521822.001.0001

[B35] Hammer R. Bar-Hillel A. Hertz T. Weinshall D. Hochstein S. (2008). Comparison processes in category learning: from theory to behavior. Brain Res. 1225, 102–118. 10.1016/j.brainres.2008.04.079 18614160

[B36] Hammer R. Diesendruck G. Weinshall D. Hochstein S. (2009a). The development of category learning strategies: what makes the difference? Cognition 112, 105–119. 10.1016/j.cognition.2009.03.012 19426967

[B37] Hammer R. Hertz T. Hochstein S. Weinshall D. (2009b). Category learning from equivalence constraints. Cogn. Process. 10, 211–232. 10.1007/s10339-008-0243-x 19050949

[B38] Higgins E. J. Ross B. H. (2011). Comparisons in category learning: how best to compare for what? in Proceedings of the 33rd Annual Conference of the Cognitive Science Society, eds Carlson L. Holscher C. Shipley T. (Austin, TX: Cogntive Science Society), 1388–1393.

[B39] Hull C. L. (1920). Quantitative aspects of evolution and concepts: an experimental study. Psychol. Monogr. 28, 1–86. 10.1037/h009313021392502

[B40] Janiszewski C. Noel H. Sawyer A. G. (2003). A meta−analysis of the spacing effect in verbal learning: implications for research on advertising repetition and consumer memory. J. Consum. Res. 30, 138–149 10.1086/374692

[B41] Jones M. Sieck W. R. (2003). Learning myopia: an adaptive recency effect in category learning. J. Exp. Psychol. Learn. Mem. Cogn. 29, 626–640. 10.1037/0278-7393.29.4.626 12924863

[B42] Kalish C. W. Rogers T. T. Lang J. Zhu X. (2011). Can semi-supervised learning explain incorrect beliefs about categories? Cognition 120, 106–118. 10.1016/j.cognition.2011.03.002 21474122

[B43] Kang S. H. K. Pashler H. (2012). Learning painting styles: spacing is advantageous when it promotes discriminative contrast. Appl. Cogn. Psychol. 26, 97–103 10.1002/acp.1801

[B44] Karpicke J. D. Bauernschmidt A. (2011). Spaced retrieval: absolute spacing enhances learning regardless of relative spacing. J. Exp. Psychol. Learn. Mem. Cogn. 37, 1250–1257. 10.1037/a0023436 21574747

[B45] Karpicke J. D. Roediger H. L. (2009). Is expanding retrieval a superior method for learning text materials? Mem. Cogn. 38, 116–124. 10.3758/MC.38.1.116 19966244

[B46] Kornell N. Bjork R. A. (2008). Learning concepts and categories: is spacing the “enemy of induction”? Psychol. Sci. 19, 585–592. 10.1111/j.1467-9280.2008.02127.x 18578849

[B47] Kornell N. Castel A. D. Eich T. S. Bjork R. A. (2010). Spacing as the friend of both memory and induction in young and older adults. Psychol. Aging 25, 498–503. 10.1037/a0017807 20545435

[B48] Krug D. Davis T. B. Glover J. A. (1990). Massed versus distributed repeated reading: a case of forgetting helping recall? J. Educ. Psychol. 82, 366–371 10.1037/0022-0663.82.2.366

[B49] Kruschke J. K. (1992). ALCOVE: an exemplar-based connectionist model of category learning. Psychol. Rev. 99, 22–44. 10.1037/0033-295X.99.1.22 1546117

[B50] Kurtz K. H. Hovland C. I. (1956). Concept learning with differing sequences of instances. J. Exp. Psychol. 51, 239–243. 10.1037/h0040295 13306871

[B51] Lee T. D. (2012). Contextual interference: generalizability and limitations BT—skill acquisition in sport: research, theory and practice, in Skill Acquisition in Sport: Research, Theory and Practice, eds Goode S. Magill R. A. (London: Routledge), 79–93.

[B52] Lee E. S. MacGregor J. N. Bavelas A. Mirlin L. (1988). The effects of error transformations on classification performance. J. Exp. Psychol. Learn. Mem. Cogn. 14, 66–74 10.1037/0278-7393.14.1.66

[B53] Li N. Cohen W. W. Koedinger K. R. (2013). Problem order implications for learning. Int. J. Artif. Intell. Educ. 23, 71–93 10.1007/s40593-013-0005-5

[B54] Lipsitt L. P. (1961). Simultaneous and successive discrimination-learning in children. Child Dev. 32, 337–347. 10.2307/1125948 13762603

[B55] Loess H. B. Duncan C. P. (1952). Human discrimination learning with simultaneous and successive presentation of stimuli. J. Exp. Psychol. 44, 215–221. 10.1037/h0061719 12990765

[B56] Logan J. M. Balota D. A. (2008). Expanded vs. equal interval spaced retrieval practice: exploring different schedules of spacing and retention interval in younger and older adults. Aging Neuropsychol. Cogn. 15, 257–280. 10.1080/13825580701322171 18421627

[B57] Love B. C. Medin D. L. Gureckis T. M. (2004). SUSTAIN: a network model of category learning. Psychol. Rev. 111, 309–332. 10.1037/0033-295X.111.2.309 15065912

[B58] MacCaslin E. F. (1954). Successive and simultaneous discrimination as a function of stimulus-similarity. Am. J. Psychol. 67, 308–314. 10.2307/1418632 13158646

[B59] Maddox W. T. Ashby F. G. Bohil C. J. (2003). Delayed feedback effects on rule-based and information-integration category learning. J. Exp. Psychol. Learn. Mem. Cogn. 29, 650–662. 10.1037/0278-7393.29.4.650 12924865

[B60] Mathy F. Feldman J. (2009). A rule-based presentation order facilitates category learning. Psychon. Bull. Rev. 16, 1050–1057. 10.3758/PBR.16.6.1050 19966254

[B61] McLaren I. P. L. Mackintosh N. J. (2002). Associative learning and elemental representation: II. Generalization and discrimination. Anim. Learn. Behav. 30, 177–200. 10.3758/BF03192828 12391785

[B62] Mitchell C. J. Nash S. Hall G. (2008). The intermixed-blocked effect in human perceptual learning is not the consequence of trial spacing. J. Exp. Psychol. Learn. Mem. Cogn. 34, 237–242. 10.1037/0278-7393.34.1.237 18194066

[B63] Mundy M. E. Honey R. C. Dwyer D. M. (2007). Simultaneous presentation of similar stimuli produces perceptual learning in human picture processing. J. Exp. Psychol. Anim. Behav. Process. 33, 124–138. 10.1037/0097-7403.33.2.124 17469961

[B64] Mundy M. E. Honey R. C. Dwyer D. M. (2008). Superior discrimination between similar stimuli after simultaneous exposure. Q. J. Exp. Psychol. 62, 18–25. 10.1080/17470210802240614 18720275

[B65] Murray J. T. (1983). Spacing Phenomena in Human Memory: a Study-Phase Retrieval Interpretation. Los Angeles, CA: University of California.

[B66] Nosofsky R. M. (1986). Attention, similarity, and the identification-categorization relationship. J. Exp. Psychol. Gen. 115, 39–61. 10.1037/0096-3445.115.1.39 2937873

[B67] Oakes L. M. Ribar R. J. (2005). A comparison of infants' categorization in paired and successive presentation familiarization tasks. Infancy 7, 85–98 10.1207/s15327078in0701_7 33430540

[B68] Pashler H. Rohrer D. Cepeda N. J. Carpenter S. K. (2007). Enhancing learning and retarding forgetting: choices and consequences. Psychon. Bull. Rev. 14, 187–193. 10.3758/BF03194050 17694899

[B69] Pyc M. A. Rawson K. A. (2009). Testing the retrieval effort hypothesis: does greater difficulty correctly recalling information lead to higher levels of memory? J. Mem. Lang. 60, 437–447 10.1016/j.jml.2009.01.004

[B70] Rawson K. A. Thomas R. C. Jacoby L. L. (2014). The power of examples: illustrative examples enhance conceptual learning of declarative concepts. Educ. Psychol. Rev. 1–22 10.1007/s10648-014-9273-3

[B71] Rehder B. Hoffman A. B. (2005). Thirty-something categorization results explained: selective attention, eyetracking, and models of category learning. J. Exp. Psychol. Learn. Mem. Cogn. 31, 811–829. 10.1037/0278-7393.31.5.811 16248736

[B72] Rickard T. C. Lau J. S. H. Pashler H. (2008). Spacing and the transition from calculation to retrieval. Psychon. Bull. Rev. 15, 656–661. 10.3758/PBR.15.3.656 18567270

[B73] Rohrer D. (2012). Interleaving helps students distinguish among similar concepts. Educ. Psychol. Rev. 24, 355–367 10.1007/s10648-012-9201-3

[B74] Rohrer D. Dedrick R. F. Burgess K. (2014a). The benefit of interleaved mathematics practice is not limited to superficially similar kinds of problems. Psychon. Bull. Rev. 21, 1323–1330. 10.3758/s13423-014-0588-3 24578089

[B75] Rohrer D. Dedrick R. F. Stershic S. (2014b). Interleaved practice improves mathematics learning. J. Educ. Psychol. 1–9. 10.1037/edu000000124578089

[B76] Rohrer D. Taylor K. (2007). The shuffling of mathematics problems improves learning. Instr. Sci. 35, 481–498 10.1007/s11251-007-9015-8

[B77] Ross B. H. (1984). Remindings and their effects in learning a cognitive skill. Cogn. Psychol. 16, 371–416. 10.1016/0010-0285(84)90014-8 6478776

[B78] Ross B. H. Kennedy P. T. (1990). Generalizing from the use of earlier examples in problem solving. J. Exp. Psychol. Learn. Mem. Cogn. 16, 42–55 10.1037//0278-7393.16.1.42

[B79] Samuels S. J. (1969). Effect of simultaneous versus successive discrimination training on paired-associate learning. J. Educ. Psychol. 60, 46–48 10.1037/h0026671

[B80] Sandhofer C. M. Doumas L. A. A. (2008). Order of presentation effects in learning color categories. J. Cogn. Dev. 9, 194–221 10.1080/15248370802022639

[B81] Schyns P. G. Rodet L. (1997). Categorization creates functional features. J. Exp. Psychol. Learn. Mem. Cogn. 23, 681–696 10.1037/0278-7393.23.3.681

[B82] Shea J. B. Morgan R. L. (1979). Contextual interference effects on the acquisition, retention, and transfer of a motor skill. J. Exp. Psychol. Hum. Learn. Mem. 5, 179–187 10.1037/0278-7393.5.2.179

[B83] Sloutsky V. M. Fisher A. V. (2004). Induction and categorization in young children: a similarity-based model. J. Exp. Psychol. Gen. 133, 166–188. 10.1037/0096-3445.133.2.166 15149249

[B84] Smith J. D. Boomer J. Zakrzewski A. C. Roeder J. L. Church B. A. Ashby F. G. (2014). Deferred feedback sharply dissociates implicit and explicit category learning. Psychol. Sci. 25, 447–457. 10.1177/0956797613509112 24335605 PMC3946254

[B85] Smith S. M. Glenberg A. Bjork R. A. (1978). Environmental context and human memory. Mem. Cogn. 6, 342–353 10.3758/BF03197465

[B86] Spiering B. J. B. J. Ashby F. G. (2008). Initial training with difficult items facilitates information integration, but not rule-based category learning. Psychol. Sci. 19, 1169. 10.1111/j.1467-9280.2008.02219.x 19076490 PMC2605282

[B87] Ste-Marie D. M. Clark S. E. Findlay L. C. Latimer A. E. (2004). High levels of contextual interference enhance handwriting skill acquisition. J. Motor Behav. 36, 115–126. 10.3200/JMBR.36.1.115-126 14766494

[B88] Stewart N. Brown G. D. A. Chater N. (2002). Sequence effects in categorization of simple perceptual stimuli. J. Exp. Psychol. Learn. Mem. Cogn. 28, 3–11. 10.1037/0278-7393.28.1.3 11831211

[B89] Taylor K. Rohrer D. (2010). The effects of interleaved practice. Appl. Cogn. Psychol. 24, 837–848 10.1002/acp.1598

[B90] Vlach H. A. (2014). The spacing effect in children's generalization of knowledge: allowing children time to forget promotes their ability to learn. Child Dev. Perspect. 8, 163–168 10.1111/cdep.12079

[B91] Vlach H. A. Ankowski A. A. Sandhofer C. M. (2012). At the same time or apart in time? The role of presentation timing and retrieval dynamics in generalization. J. Exp. Psychol. Learn. Mem. Cogn. 38, 246–254. 10.1037/a0025260 21895392 PMC3302959

[B92] Wahlheim C. N. Dunlosky J. Jacoby L. L. (2011). Spacing enhances the learning of natural concepts: an investigation of mechanisms, metacognition, and aging. Mem. Cogn. 39, 750–763. 10.3758/s13421-010-0063-y 21264639 PMC3085105

[B93] Wahlheim C. N. Finn B. Jacoby L. L. (2012). Metacognitive judgments of repetition and variability effects in natural concept learning: evidence for variability neglect. Mem. Cogn. 40, 703–716. 10.3758/s13421-011-0180-2 22282159

[B94] Wahlheim C. N. Maddox G. B. Jacoby L. L. (2014). The role of reminding in the effects of spaced repetitions on cued recall: sufficient but not necessary. J. Exp. Psychol. Learn. Mem. Cogn. 40, 94–105. 10.1037/a0034055 23937236 PMC4032790

[B95] Whitman J. R. Garner W. R. (1963). Concept learning as a function of form of internal structure. J. Verbal Learn. Verbal Behav. 2, 195–202 10.1016/S0022-5371(63)80085-7

[B96] Williams J. P. Ackerman M. D. (1971). Simultaneous and successive discrimination of similar letters. J. Educ. Psychol. 62, 132–137. 10.1037/h00307821235793

[B97] Xue G. Dong Q. Chen C. Lu Z. Mumford J. A. Poldrack R. A. (2010). Greater neural pattern similarity across repetitions is associated with better memory. Science 330, 97–101. 10.1126/science.1193125 20829453 PMC2952039

[B98] Zulkiply N. Burt J. S. (2013). The exemplar interleaving effect in inductive learning: moderation by the difficulty of category discriminations. Mem. Cogn. 41, 16–27. 10.3758/s13421-012-0238-9 22886736

[B99] Zulkiply N. McLean J. Burt J. S. Bath D. (2012). Spacing and induction: application to exemplars presented as auditory and visual text. Learn. Instr. 22, 215–221 10.1016/j.learninstruc.2011.11.002

