# Supplementary material for: What you learn is more than what you see: what can sequencing effects tell us about inductive category learning?
Source: Front Psychol. 2015 Apr 30;6:505. doi: 10.3389/fpsyg.2015.00505 (PMC4415402; doi:10.3389/fpsyg.2015.00505)
Supplement: Supplementary file 1 [file Table1.DOCX]

Table 1

*Details of a survey of 51 studies comparing interleaved and blocked sequences of study. The studies included in this survey used different types of tasks and types of stimuli organization. The repetition ratio was calculated by dividing the number of items presented during study by the total number of categories used for studies using stimuli organized by categories. The type of stimuli was evaluated by visually inspecting the stimuli presented by the authors or the description available in the paper.*

| **Source** | **Experiment** | **Participants** | **Stimuli** | **Better Sequence?** | **Type of Stimuli** | **Type of Task** | **Number of Categories** | **Number of Items per category** | **Repetition Ratio** |
| --- | --- | --- | --- | --- | --- | --- | --- | --- | --- |
| Birnbaum, M. S., Kornell, N., Bjork, E. L., & Bjork, R. A. (2013). | Experiment 1 | Adults | Birds | Simultaneous and Interleaved | Low Discriminability | Categorization | 8 | 4 | 0.5 |
| Birnbaum, M. S., Kornell, N., Bjork, E. L., & Bjork, R. A. (2013). | Experiment 2 | Adults | Butterflies | Interleaved better than all. Spaced (both versions) better than blocked | Low Discriminability | Categorization | 8 | 4 | 0.5 |
| Carpenter, S. K., & Mueller, F. E. (2013) | Experiment 1 | Adults | French Pronunciation | Blocked | High Discriminability | Categorization | 8 | 4 | 0.5 |
| Carpenter, S. K., & Mueller, F. E. (2013). | Experiment 2 | Adults | French Pronunciation | Blocked | High Discriminability | Recall | 8 | 4 | 0.5 |
| Carpenter, S. K., & Mueller, F. E. (2013). | Experiment 2 | Adults | French Pronunciation | Blocked | High Discriminability | Categorization | 5 | 15 | 3 |
| Carvalho, P. F. & Albuquerque (2012). | Experiment 1 | Adults | Checkerboards | Interleaved | Low Discriminability | Discrimination | Not organized in categories | - | - |
| Carvalho, P. F., & Goldstone, R. L. (2014b). | Experiment 1 | Adults | High similarity blob categories | Interleaved | Low Discriminability | Categorization | 3 | 8 | 2.666666667 |
| Carvalho, P. F., & Goldstone, R. L. (2014b). | Experiment 1 | Adults | Low similarity blob categories | Blocked | High Discriminability | Categorization | 3 | 8 | 2.666666667 |
| Carvalho, P. F., & Goldstone, R. L. (2014c). | Experiment 1 & 2 | Adults | High similarity novel categories | Interleaved | Low Discriminability | Active Categorization | 3 | 4 | 1.333333333 |
| Carvalho, P. F., & Goldstone, R. L. (2014c). | Experiment 1 & 2 | Adults | High similarity novel categories | Blocked | Low Discriminability | Passive Categorization | 3 | 4 | 1.333333333 |
| Dwyer, D. M., & Vladeanu, M. (2009). | Experiment 2 | Adults | 3D face images morphs | Interleaved similar | Low Discriminability | Discrimination | Not organized in categories | - | - |
| Dwyer, D. M., Hodder, K. I., & Honey, R. C. (2004). | Experiment 1-2 | Adults | Flavor Compounds | Interleaved | Low Discriminability | Discrimination | Not organized in categories | - | - |
| Dwyer, D. M., Mundy, M. E., & Honey, R. C. (2011). | Experiment 1-2 | Adults | Faces and checkerboards | Interleaved | Low Discriminability | Discrimination | Not organized in categories | - | - |
| Gagné, R. M. (1950). | Only one experiment | Adults | pairings figure-non-sense word | Blocked | High Discriminability | Pair-association | 4 | 3 | 0.75 |
| Goldstone, R. L. (1996). | Experiment 2 | Adults | Novel objects | Blocked | High Discriminability | Categorization | 2 | 300 | 150* |
| Hall, K. G., Domingues, D. A., & Cavazos, R. (1994). | Only one experiment | Adults | Motor responses | Interleaved | Low Discriminability | Motor response acquisition | 3 | 15 | 5 |
| Higgins, E. J., & Ross, B. H. (2011) | Experiment 2 | Adults | Birds | Interleaved | Low Discriminability | Categorization | 6 | 6 | 1 |
| Higgins, E. J., & Ross, B. H. (2011). | Experiment 3 | Adults | Math principles (permutations and combinations) | Blocked | High Discriminability | Categorization | 2 | 2 | 1 |
| Kang, S. H. K., & Pashler, H. (2012). | Experiment 1 | Adults | Paintings | Interleaved | Low Discriminability | Categorization | 3 | 24 | 8 |
| Kang, S. H. K., & Pashler, H. (2012). | Experiment 2 | Adults | Paintings | Interleaved and Simultaneous Different | Low Discriminability | Categorization | 3 | 10 | 3.333333333 |
| Kornell, N., & Bjork, R. A. (2008) | Experiment 1A | Adults | Paintings | Interleaved | Low Discriminability | Categorization | 12 | 6 | 0.5 |
| Kornell, N., & Bjork, R. A. (2008). | Experiment 1B | Adults | Paintings | Interleaved | Low Discriminability | Categorization | 12 | 6 | 0.5 |
| Kornell, N., & Bjork, R. A. (2008). | Experiment 2 | Adults | Paintings | Interleaved | Low Discriminability | Memory for Category | 12 | 6 | 0.5 |
| Kornell, N., Castel, A. D., Eich, T. S., & Bjork, R. A. (2010) | Only one experiment | Adults | Paintings | Interleaved | Low Discriminability | Categorization | 6 | 6 | 1 |
| Kurtz, K. H., & Hovland, C. I. (1956). | Only one experiment | Adults | Novel objects | Blocked | High Discriminability | Categorization | 4 | 8 | 2 |
| Lavis, Y., & Mitchell, C. J. (2006) | Experiment 2A | Adults | Checkerboards | Interleaved | Low Discriminability | Discrimination | Not organized in categories | - | - |
| Lavis, Y., & Mitchell, C. J. (2006). | Experiment 1A | Adults | Checkerboards | Interleaved | Low Discriminability | Categorization | 2 | 2 | 1 |
| Lavis, Y., & Mitchell, C. J. (2006). | Experiment 1B | Adults | Checkerboards | Interleaved | Low Discriminability | Discrimination | Not organized in categories | - | - |
| Mitchell, C. J., Kadib, R., Nash, S., Lavis, Y., & Hall, G. (2008). | Experiment 1A | Adults | Checkerboards | Interleaved | Low Discriminability | Discrimination | Not organized in categories | - | - |
| Mitchell, C. J., Kadib, R., Nash, S., Lavis, Y., & Hall, G. (2008). | Experiment 1B | Adults | Checkerboards | Interleaved | Low Discriminability | Discrimination | Not organized in categories | - | - |
| Mitchell, C. J., Kadib, R., Nash, S., Lavis, Y., & Hall, G. (2008). | Experiment 2 | Adults | Checkerboards | Interleaved | Low Discriminability | Discrimination | Not organized in categories | - | - |
| Mitchell, C. J., Kadib, R., Nash, S., Lavis, Y., & Hall, G. (2008). | Experiment 3 | Adults | Checkerboards | Interleaved | Low Discriminability | Discrimination | Not organized in categories | - | - |
| Mitchell, C. J., Kadib, R., Nash, S., Lavis, Y., & Hall, G. (2008). | Experiment 4 | Adults | Checkerboards | Interleaved similar | Low Discriminability | Discrimination | Not organized in categories | - | - |
| Mitchell, C. J., Nash, S., & Hall, G. (2008). | Experiment 1 | Adults | Checkerboards | Interleaved | Low Discriminability | Discrimination | Not organized in categories | - | - |
| Mitchell, C. J., Nash, S., & Hall, G. (2008). | Experiment 2 | Adults | Checkerboards | Interleaved | Low Discriminability | Discrimination | Not organized in categories | - | - |
| Mundy, M. E., Honey, R. C., & Dwyer, D. M. (2007). | Experiment 2 | Adults | Face morphs | Interleaved | Low Discriminability | Discrimination | Not organized in categories | - | - |
| Mundy, M. E., Honey, R. C., & Dwyer, D. M. (2008). | Experiment 1 | Adults | Checkerboards | Interleaved | Low Discriminability | Discrimination | Not organized in categories | - | - |
| Rohrer, D., & Taylor, K. (2007). | Experiment 2 | Adults | Math principles (volume of geometric solids) | Interleaved | Low Discriminability | Solving problems | 4 | 4 | 1 |
| Sandhofer, C. M., & Doumas, L. A. A. (2008). | Experiment 1 | Children | Objects and color names | Blocked | High Discriminability | Categorization AND Memory | 3 | 6 | 2 |
| Shea, J. B., & Morgan R. L. (1979). | Only one experiment | Adults | Motor responses | Interleaved | Low Discriminability | Motor response acquisition | 3 | 18 | 6 |
| Ste-Marie, D. M., Clark, S. E., Findlay, L. C., & Latimer, A. E. (2004). | Experiment 1 | 1st grade children | Handwriting | Interleaved | Low Discriminability | Production/memory | 3 | 24 | 8 |
| Ste-Marie, D. M., Clark, S. E., Findlay, L. C., & Latimer, A. E. (2004). | Experiment 2 | 1st grade children | Handwriting | interleaved (mostly) | Low Discriminability | Production/memory | 3 | 24 | 8 |
| Ste-Marie, D. M., Clark, S. E., Findlay, L. C., & Latimer, A. E. (2004). | Experiment 3 | 1st grade children | Handwriting | interleaved | Low Discriminability | Production/memory | 3 | 24 | 8 |
| Taylor, K., & Rohrer, D. (2010). | Only one experiment | Children | Math principles (volume of geometric solids) | Interleaved | Low Discriminability | Solving problems | 4 | 4 | 1 |
| Wahlheim, C. N., Dunlosky, J., & Jacoby, L. L. (2011). | Experiment 1 & 2 | Adults | Birds | Interleaved | Low Discriminability | Categorization | 6 | 6 | 1 |
| Whitman, J. R., & Garner, W. R. (1963). | Only one experiment | Adults | Relation in shape figures | Blocked | High Discriminability | Categorization | 2 | 8 | 4 |
| Zulkiply, N., & Burt, J. S. (2012). | Experiment 1 | Adults | Paintings | Interleaved | Low Discriminability | Categorization | 12 | 6 | 0.5 |
| Zulkiply, N., & Burt, J. S. (2012). | Experiment 2 | Adults | Complex objects (low discriminability) | Interleaved | Low Discriminability | Categorization | 12 | 10 | 0.833333333 |
| Zulkiply, N., & Burt, J. S. (2012). | Experiment 2 | Adults | Complex objects (high discriminability) | Blocked | High Discriminability | Categorization | 12 | 10 | 0.833333333 |
| Zulkiply, N., McLean, J., Burt, J. S., & Bath, D. (2012). | Experiment 1 | Adults | Text descriptions (written) | Interleaved | Low Discriminability | Categorization | 6 | 3 | 0.5 |
| Zulkiply, N., McLean, J., Burt, J. S., & Bath, D. (2012). | Experiment 2 | Adults | Text descriptions (listened) | Interleaved | Low Discriminability | Categorization | 6 | 3 | 0.5 |
